# Aging of the Peritoneal Dialysis Membrane

**DOI:** 10.3389/fphys.2022.885802

**Published:** 2022-04-22

**Authors:** Raymond T. Krediet

**Affiliations:** Division of Nephrology, Department of Medicine, Amsterdam University Medical Center, Amsterdam, Netherlands

**Keywords:** peritoneal dialysis, ultrafiltration failure, glucose, pseudohypoxia, GLUT-1, growth factors, osmotic agents

## Abstract

Long-term peritoneal dialysis as currently performed, causes structural and functional alterations of the peritoneal dialysis membrane. This decay is brought about by the continuous exposure to commercially available glucose-based dialysis solutions. This review summarizes our knowledge on the peritoneum in the initial phase of PD, during the first 2 years and the alterations in function and morphology in long-term PD patients. The pseudohypoxia hypothesis is discussed and how this glucose-induced condition can be used to explain all peritoneal alterations in long-term PD patients. Special attention is paid to the upregulation of hypoxia inducing factor-1 and the subsequent stimulation of the genes coding for glucose transporter-1 (GLUT-1) and the growth factors transforming growth factor-β (TGFβ), vascular endothelial growth factor (VEGF), plasminogen growth factor activator inhibitor-1 (PAI-1) and connective tissue growth factor (CTGF). It is argued that increased pseudohypoxia-induced expression of GLUT-1 in interstitial fibroblasts is the key factor in a vicious circle that augments ultrafiltration failure. The practical use of the protein transcripts of the upregulated growth factors in peritoneal dialysis effluent is considered. The available and developing options for prevention and treatment are examined. It is concluded that low glucose degradation products/neutral pH, bicarbonate buffered solutions with a combination of various osmotic agents all in low concentration, are currently the best achievable options, while other accompanying measures like the use of RAAS inhibitors and tamoxifen may be valuable. Emerging developments include the addition of alanyl glutamine to the dialysis solution and perhaps the use of nicotinamide mononucleotide, available as nutritional supplement.

## Introduction

The use of the peritoneum as a dialysis membrane in patients with end-stage renal disease (ESRD) for the removal of solutes and fluid excess implies that the feasibility of this mode of chronic renal replacement therapy is dependent on one single membrane. Anything used intensively will decay in the long-term and the peritoneum of patients treated with peritoneal dialysis (PD) is no exception. It is however likely that the decline rate can be retarded by appropriate modifications of the PD treatment. In this review the morphological alterations and their likely causes will be discussed and their impact on peritoneal transport. Special attention will be given to glucose-induced pseudohypoxia and its effects on various growth factors and glucose-transporter-1 expression. The effects of prophylaxis and treatment, especially by adjustments of the dialysis solutions will also be examined.

## The Peritoneum in the Initial Phase of PD

The peritoneal membrane is not a single structure, but comprises the mesothelial layer and the submesothelial interstitial layer, in which blood and lymphatic vessels are dispersed. The interstitium is composed of a ground substance consisting of hyaluronan and glycosaminoglycans, and a fibrous collagen network that constitutes a scaffold for the embedded structures. Adipose cells and a few fibroblasts are the predominant cell types. The mesothelial layer offers no hindrance to peritoneal transport (Flessner, 1994), meaning that the volume of distribution of the instilled dialysis fluid includes both the peritoneal cavity and the interstitium. Consequently the peritoneal microcirculatory blood vessels are the determinants of peritoneal solute and fluid transport from the circulation to the dialysate-filled peritoneal cavity. Transport across the microvascular wall occurs through a system of pores (Pappenheimer et al., 1951). According to the generally accepted 3-pore theory solute transfer during PD occurs through inter-endothelial pores, but additionally water is also transported by the intra-endothelial water channel aquaporin-1 (AQP-1) (Rippe and Stelin, 1989; Rippe, 1993). Small inter-endothelial pores with a radius of about 40 Ǻ constitute 90% of the total number of pores and allow the transport of small solutes that all have radii < 3 Ǻ and low molecular weight proteins like β_2_-microglobulin. Small clefts between endothelial cells are the most probable morphological equivalent of the small pores. Transport through these is by diffusion to the dialysate-filled interstitium. The contribution of convection is small for low molecular weight solutes, due to the low hydrostatic pressure gradient and the high diffusion rates of these solutes. The low hydrostatic pressure gradient does however induce a small amount of fluid transport, similar to trans-capillary ultrafiltration in the non-PD situation. In addition to the abundance of small pores, a limited quantity of large inter-endothelial pores is located in the venular part of the microcirculation. Their radii exceed 150 Ǻ and they allow the passage of large molecules like serum proteins. As water removal from the circulation by hydrostatic filtration is limited and counteracted by back-filtration into the circulation by colloid osmosis, PD as kidney replacement therapy is only possible by the creation of a crystalloid osmotic pressure gradient. This is usually achieved by the addition of large quantities of glucose (molecular weight 180 Da, radius < 3 Ǻ) to the dialysis solution. The small size of the glucose molecule implies that it is not very efficient as an osmotic agent, because after 4 h about 60% is reabsorbed into the circulation (Smit et al., 2003). Consequently any efficacy for fluid transport through the small pores is only present during the first few hours of a dialysis dwell (Parikova et al., 2008). The presence of the water channel AQP-1 in peritoneal capillaries and venules explains the water removal by glucose, because only water can traverse this water channel. The radius of AQP-1 is too small to enable the passage of solutes, meaning that it allows free water transport by a very efficient crystalloid osmotic gradient, irrespective of the osmole employed (Ni et al., 2006).

Already at the start of PD the inter-individual variation of peritoneal transport parameters is very large (Twardowski et al., 1987). This may partly be related to differences in the genotype of various factors or structures involved in peritoneal transport. For instance the inflammatory cytokine interleukin-6 (IL-6) is locally produced in the peritoneal cavity during PD and has a number of polymorphisms. It appeared that the CC genotype had the highest expression in peritoneal effluent and also the highest concentration of the protein, which was associated with a faster solute transport compared to the other genotypes (Gillerot et al., 2005). However follow-up to 36 months failed to show any influence of IL-6 polymorphisms on peritoneal transport (Lee et al., 2011), suggesting that local PD-related effects become more important with PD duration.

## The Peritoneal Membrane in the First Two Years of PD

Alterations in peritoneal morphology and transport occur already during the first few years of PD. After 3 months advanced glycosylation end-products (AGEs) -formed from non-enzymatic interactions between glucose and amino acid residues and leading to irreversible cross linking of tissue proteins-can be found in the mesothelial and submesothelial layer (Yamada et al., 1994). Endothelial-to-mesenchymal transition of mesothelial cells (mesothelial-to-mesenchymal transition, MMT) is the earliest morphological change. This phenomenon has first been described in 2003 and consists of a transition of the usual epithelial phenotype to a mesenchymal one with loss of cytokeratin expression. MMT in peritoneal biopsies is characterized by the presence of cytokeratin-positive fibroblasts-like cells in the submesothelial interstitial tissue (Yáñez-Mó et al., 2003). The prevalence of MMT is highest between 1.5 and 2 years, when it is present in about one third of patients (Del Peso et al., 2008) The cytokeratin-positive fibroblasts produce large amounts of vascular endothelial growth factor (VEGF) (Aroeira et al., 2005). MMT is accompanied by high effluent concentrations of the mesothelial cell marker cancer antigen 125 (CA125) and by high small solute transfer rates. It may explain the relationship between the creatinine transport rate and both effluent CA125 and VEGF in incident PD patients who never had peritonitis, during the first year of treatment (van Esch et al., 2004).

MMT occurs during the first 2 years of PD and has been claimed to predict long-term peritoneal membrane alterations, but this is only a hypothesis in the absence of follow-up biopsies.

Vascular density in the interstitium is not different from that before the start of PD, but the wall/total surface ratio of small arteries is somewhat increased compared to non-dialyzed patients with end-stage kidney failure (Mateijsen et al., 1999). This has been called vasculopathy and was present in a mild form in 20–30% of patients during the first 2 years (Williams et al., 2002; Del Peso et al., 2008). About 40% of peritoneal capillaries was of the immature type, i.e., stained negative for α-smooth muscle actin (α-SMA) (Nakano et al., 2020).

Longitudinal follow-up of peritoneal transport either showed no effect on peritoneal solute transport and ultrafiltration (Selgas et al., 1994; Coester et al., 2014), or some increase of the dialysate/plasma (D/P) ratio of creatinine accompanied by a decrease in ultrafiltration, due to a faster disappearance of the glucose-induced osmotic gradient (Davies, 2004). Accordingly an inverse relationship was present between D/P creatinine and ultrafiltration volume during the first years on PD (Davies et al., 1993). Three possibilities could explain the reported high solute transfer rate. These include 1) the CC genotype of interleukin-6, 2) a high production of VEGF during MMT, or 3) the number of newly formed immature capillaries, because of the reported relationship with D/P creatinine (Nakano et al., 2020).

## Alterations in Peritoneal Morphology and Function in Long-Term PD

All constituents of the peritoneal dialysis membrane are affected by PD duration. Although no longitudinal studies on the development of the alterations have been published in adult PD patients treated with conventional PD solutions, a general picture can be discerned from the cross-sectional analyses, as reviewed recently (Parikova et al., 2021). Lesions that progress with PD duration include accumulation of AGEs in the subendothelial and perivascular regions. This is accompanied by loss of mesothelial cells, vasculopathy leading to subendothelial hyalinosis and narrowing of vascular lumina or even obstruction, and by an increased thickness of the submesothelial fibrous layer or even more general interstitial fibrosis of the stroma. The interstitial fibrosis consists of myofibroblasts. Vascular density is only increased in the presence of severe interstitial alterations (Mateijsen et al., 1999; Williams et al., 2002).

All longitudinal studies showed a progressive increase of peritoneal small solute transfer after 2 years (Selgas et al., 1994; Davies, 2004; Coester et al., 2014). This was accompanied by a lower ultrafiltration, which was directly related to solute transport up to 4 years of treatment. Thereafter a dissociation occurred between both parameters: ultrafiltration was markedly lower, than predicted from D/P creatinine (Davies, 2004). Analysis of ultrafiltration at 60 min after instillation of a 3.86% glucose dialysis solution showed a decrease of both small-pore fluid transport and free water transport after 4 years (Coester et al., 2014). The lower rate of small-pore fluid transport suggests a reduction of the hydrostatic pressure gradient. AGE-induced vasculopathy is the most likely cause, because the presence of a luminal stenosis likely diminishes the post-stenotic filtration pressure (Krediet, 2018; Krediet et al., 2019). Free water transport after 4 years reached its lowest values in patients who developed encapsulating peritoneal sclerosis (EPS) (Sampimon et al., 2011) and a value of less than 75 ml after a 60 min dwell was even predictive of EPS with a sensitivity of 100% and a specificity of 81% (Barreto et al., 2019).

All EPS patients had a normal expression of AQP-1 (Morelle et al., 2015). Therefore peritoneal interstitial fibrosis may be important in the observed reduction of free water transport with PD duration (Krediet et al., 2015). Two recent studies are supportive of this hypothesis. Kinetic modelling of peritoneal transport was used in a limited number of PD patients with ultrafiltration failure in the first study. The results suggested that the disappearance of glucose from the dialysate consisted of a vascular- and an interstitial component (Stachowska-Pietka et al., 2019). The tissue diffusivity of glucose appeared higher in the patients with ultrafiltration failure, than in those without this complication. This tissue diffusivity was modelled, not measured, meaning that other mechanisms like uptake in interstitial cells, cannot be excluded The second study comprised longitudinal follow-up of a large patient group treated with conventional PD solutions. A break-point in the time-course of the mass transfer area coefficient of creatinine was present after 3 years. This was also present for the percentage of the instilled glucose quantity, that had disappeared from the dialysate. (Van Diepen et al., 2020). However the slope of the increase after the breakpoint was much steeper for glucose than for creatinine. These two studies point to a contribution of interstitial fibrosis to the enhanced disappearance of glucose from the dialysis solution reducing the crystalloid osmotic gradient for AQP-1 and thereby inducing less free water transport.

**FIGURE 1:**
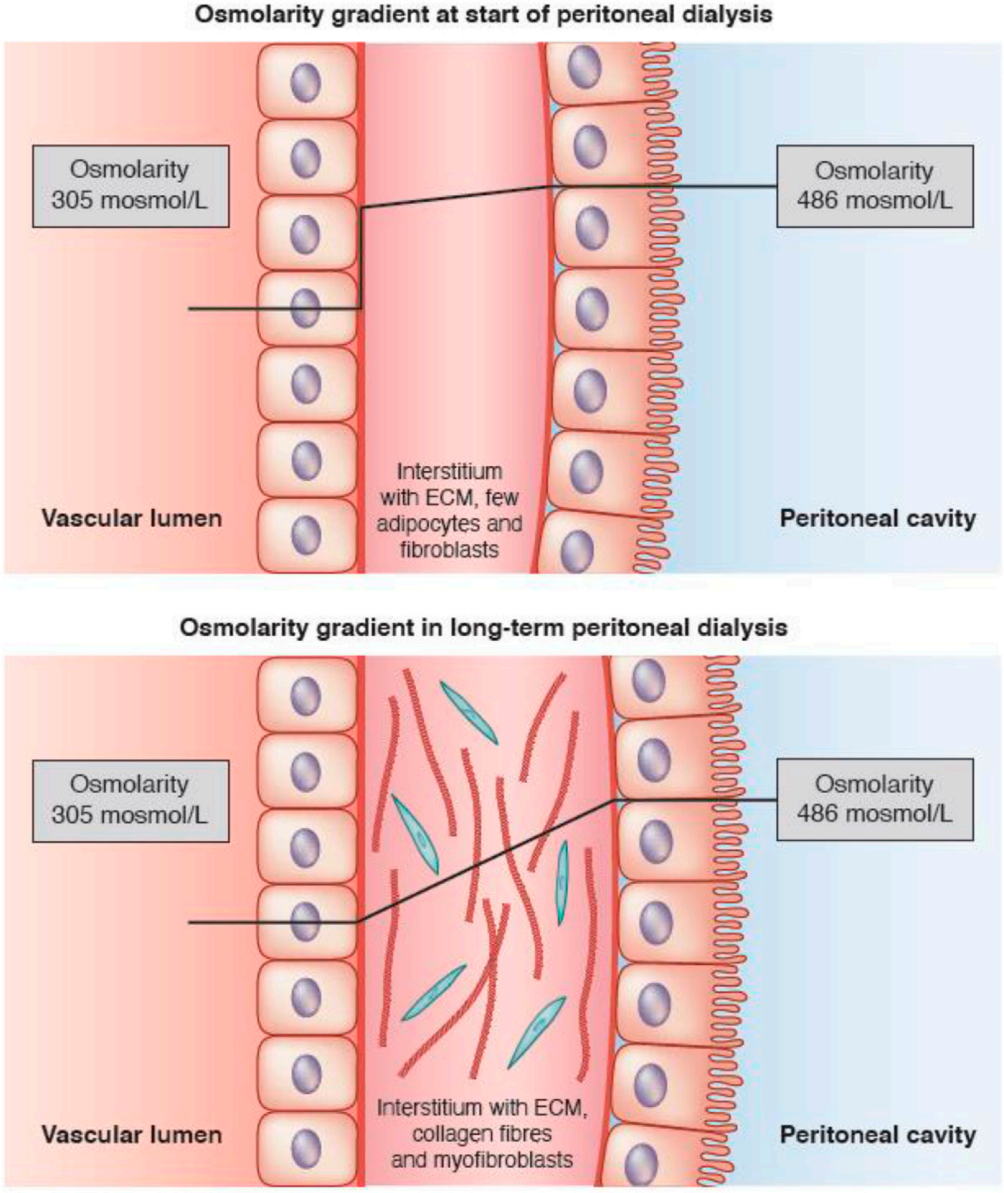
Schematic representation of the crystalloid osmotic gradient across the peritoneal interstitium in the first few years of peritoneal dialysis, when only a small amount of fibroblasts is present (upper panel), and the situation in long-term peritoneal dialysis, where the gradient decreases in the interstitium due to cellular uptake of glucose (lower panel). Aquaporin-1 (AQP-1) is present in some endothelial cells. The lines show the estimated time-course of the crystalloid osmotic pressure gradient between the dialysate-filled peritoneal cavity and the blood. Note that the pressure gradient remains almost stable during the passage of fluid through the interstitium in patients at the start of PD, resulting in a high gradient for AQP-1 (upper panel), but decreases in long-term PD patients due to uptake of glucose by interstitial myofibroblasts, leading to a lower crystalloid osmotic pressure gradient (lower panel). Taken from Krediet (2021) with permission of the American Society of Nephrology.

A glucose molecule is too large to enter a cell by simple diffusion, but requires the presence of specific proteins in the cell membrane, known as glucose transporters. Two types can be distinguished: those that facilitate diffusion (GLUTs) and sodium glucose linked transporters (SGLTs), in which cellular glucose uptake is coupled to that of sodium (Navale and Paranjape, 2016). GLUT-1 is present on the plasma membrane of many cell types including murine fibroblasts (Ortiz and Haspel, 1993) and is especially upregulated when a high cellular influx of glucose is required, for instance during hypoxia (Airley et al., 2001). It can be hypothesized that expression of GLUT-1 by peritoneal interstitial myofibroblasts, the density of which increases in long-term PD, explains the high tissue apparent diffusivity of glucose causing its enhanced disappearance from the peritoneal cavity. Both will lead to a reduction of the crystalloid osmotic gradient for AQP-1 (Krediet, 2021). This is illustrated in Figure 1.

## The Pseudohypoxia Hypothesis

Twenty years ago I suggested that glucose exposure causes a status of peritoneal tissues called pseudohypoxia analogous to the pathogenesis of diabetic complications (Krediet et al., 2002). Pseudohypoxia in diabetes mellitus has been described as a consequence of hyperglycemia (Williamson et al., 1993). An impaired oxidation of cellular nicotinamide dinucleotide (NADH) to NAD^+^ causes an increase in the NADH/NAD^+^ ratio, which is characteristic of hypoxia caused by a lack of oxygen, because this blunts NADH oxidation. The consequences in diabetics include increased superoxide anion production and possibly nitric oxide formation. Pseudohypoxia in diabetes mellitus develops, because hyperglycemia increases the influx of glucose into cells, where it is subsequently metabolized in the glycolysis to pyruvate. NAD^+^ is converted to NADH during this breakdown of glucose. Pyruvate is taken-up in the mitochondria were it contributes to the formation of acetylCoA. This product is part of the Krebs circle, which participates in the respiratory chain. This oxidation requires the donation of electrons for which NADH is one of the sources, leading to NAD^+^ regain. In case of hypoxia the conversion of NADH to NAD^+^ can to some extent be accomplished by lactate dehydrogenase, which converts pyruvate to lactate. NAD^+^ is regained during this reaction. In case of a severe hyperglycemia, intracellular glucose is also metabolized in the sorbitol pathway. The formed sorbitol is subsequently metabolized to fructose. During the latter reaction NAD^+^ is converted to NADH. In consequence the NADH/NAD^+^ increases both in the glycolysis and the sorbitol pathway, but in contrast to the glycolysis the formed NADH cannot be oxidized to NAD^+^ in the sorbitol pathway. The resulting high cytosolic NADH/NAD^+^ ratio is very similar to the situation in hypoxia or ischemia and is therefore known as pseudohypoxia.

**FIGURE 2:**
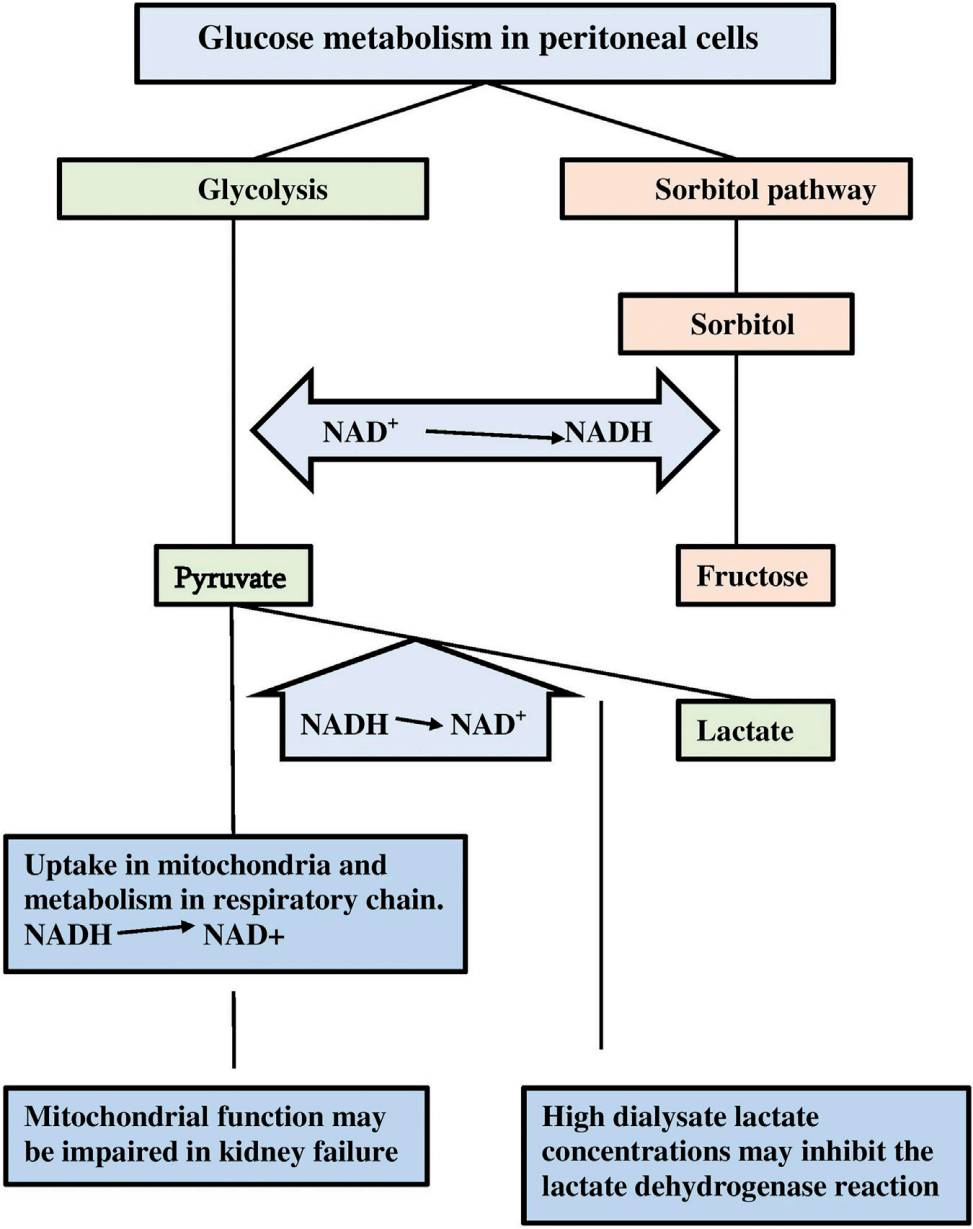
A scheme of the intracellular glucose metabolism in case of a large supply. Note the formation of NADH during the glycolysis and the sorbitol/polyol pathway, and the possible inhibition of compensatory NAD^+^ back gain due to inhibition of lactate dehydrogenase and mitochondrial dysfunction.

Non-diabetic PD patients have normal plasma glucose concentrations, but those in peritoneal dialysate exceed even severe hyperglycemic levels by far. Furthermore, some compensatory mechanisms for the oxidation of NADH to NAD^+^ may be impaired. For instance, mitochondrial dysfunction can occur in patients with kidney failure (Galvan et al., 2017). Also the use of high lactate concentrations as buffer substance in dialysis solutions may inhibit the lactate dehydrogenase activity, because extracellular lactate can traverse the cell membrane, leading to high cytosolic lactate concentrations (Halestrap and Wilson, 2012). A comparison between lactate–buffered and bicarbonate-buffered dialysis solutions aimed to compare the degree of pseudohypoxia, has never been published. The pathways for intracellular glucose metabolism in PD patients are illustrated in Figure 2.

Strong evidence for the presence of peritoneal pseudohypoxia in long-term PD patients has been published by the group of Devuyst et al. The authors reported on the expression of the genes for nitric oxide synthase and VEGF in peritoneal tissue of patients (Combet et al., 2000). The expression of both increased with PD duration (Pseudo)hypoxia enhances these compensatory vasodilating mechanisms, as has been shown for VEGF (Ferrara, 1995), which is not only produced during MMT of mesothelial cells, but local production of the effluent VEGF protein has also been shown in a cross-sectional analysis in prevalent PD patients (Zweers et al., 1999). Furthermore, an increase of effluent VEGF has been shown during longitudinal follow-up (Zweers et al., 2001). These results are all in agreement with peritoneal hypoxia in PD patients.

Hypoxia characterized by a high NADH/NAD^+^ ratio, stimulates upregulation of the hypoxia inducible factor-1 (HIF-1), which leads to upregulation of various factors, like EPO, GLUT-1, VEGF and various profibrotic and angiogenic factors (Darby and Hewitson, 2016). The latter include TGFβ, plasminogen activator inhibitor-1 (PAI-1) and connective tissue growth factor (CTGF). Increased expression of GLUT-1 on peritoneal interstitial myofibrobasts may cause a vicious circle, in which the cellular uptake of dialysate glucose is stimulated leading to more pseudohypoxia and thereby more stimulation of GLUT-1 expression, This induces a further augmentation of cellular glucose uptake and a reduction of the interstitial glucose gradient. A progressive decline in peritoneal ultrafiltration is the clinically relevant result (Krediet, 2021).

Stimulation of TGFβ, PAI-1 and CTGF by glucose-induced pseudohypoxia occurs during peritoneal dialysis and is likely relevant for the development of the peritoneal alterations that occur in long-term PD patients (Krediet and Parikova, 2022). The upregulation of TGFβ is probably most important in the development of interstitial fibrosis (Margetts et al., 2001). However the concentration of the TGFβ protein in peritoneal effluent does not reflect its biological function, because it is bound to α_2_-macroglobulin in the circulation (Zweers et al., 1999). Both PAI-1 and CTGF are downstream regulators of the TGFβ pathway. The dialysate concentrations of these pro-fibrotic proteins increase with the duration of peritoneal dialysis (Mizutani et al., 2010; Barreto et al., 2013). An effluent PAI-1 appearance rate exceeding 8.5 ng/ml had a sensitivity of 100% for a clinical diagnosis of EPS within 1 year and a specificity of 55% (Barreto et al., 2019).

**FIGURE 3:**
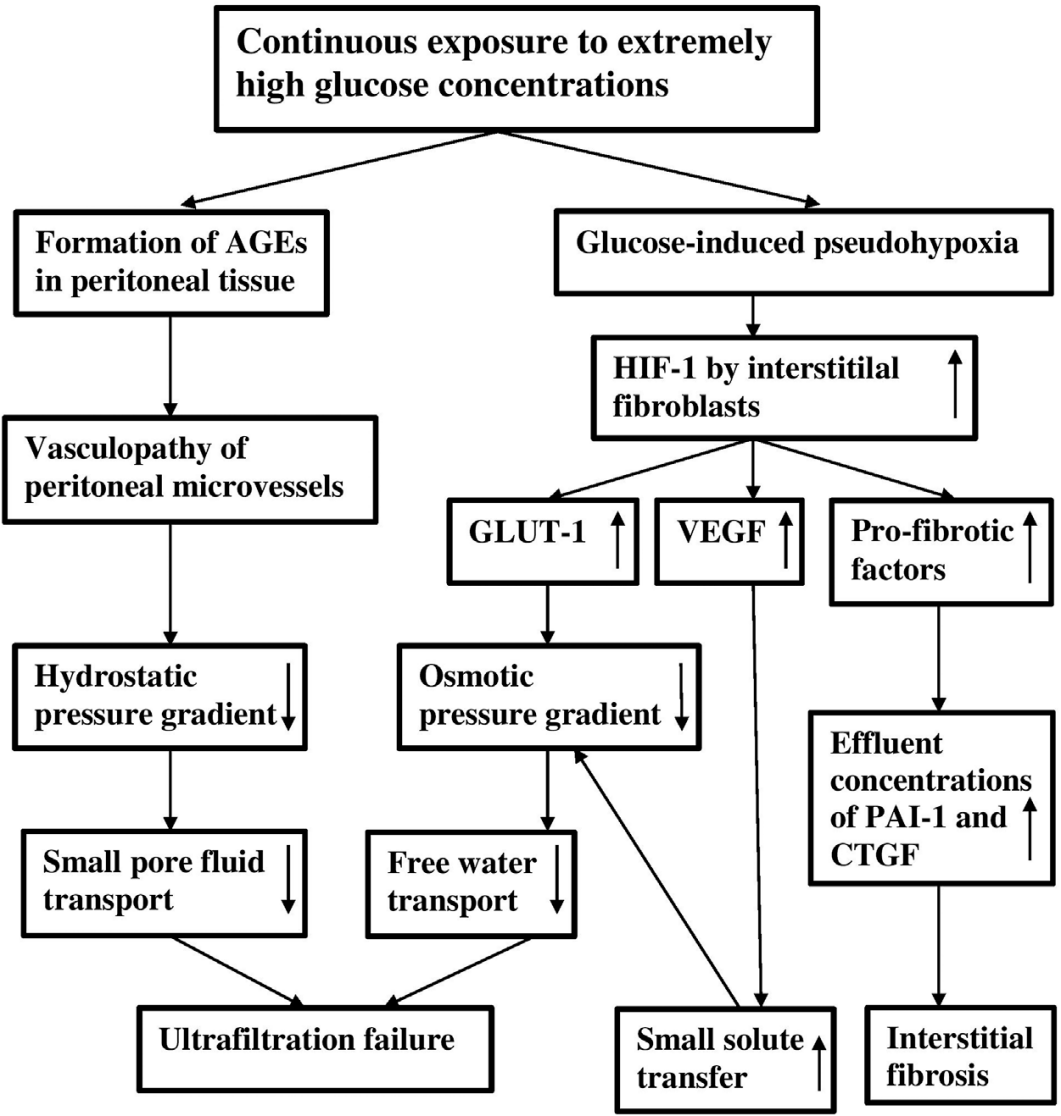
A scheme of the effects of continuous exposure to extremely high glucose concentrations on the peritoneal dialysis membrane. Note that ultrafiltration failure can be caused by a vasculopathy-induced decreased hydrostatic pressure gradient and by a decreased osmotic pressure gradient, due to increased GLUT-1 expression and to increased small solute transfer. The low osmotic pressure gradient causes a reduction of free water transport.

It can therefore be concluded that extremely high glucose concentrations in peritoneal dialysis solutions have two effects. First they lead to the formation of AGEs, that are likely involved in the genesis of vasculopathy inducing a decline of small-pore fluid transport, and second they cause pseudohypoxia, which stimulates various genes that are involved in the peritoneal alterations that occur in long-term PD patients, of which the decline in free water transport and peritoneal fibrosis are most relevant. The severe ultrafiltration failure in long-term PD patients is explained by a combination of severely impaired small-pore fluid transport and free water transport, as illustrated in Figure 3.

## Prevention and Treatment

Reduction of peritoneal exposure to the excessively high glucose concentrations is the cornerstone of pseudohypoxia prevention in peritoneal tissues and its consequences for peritoneal morphology and transport. This can be accomplished by modifications of the dialysis solutions and possibly by drugs that inhibit the causative factors for the development of the morphological and functional alterations. Modifications of the dialysis solutions consist of changes of osmotic agents, combinations of osmotic agents, changes in the dialysate buffer and additives to the solutions Also when the consequences of pseudohypoxia are already present, treatment consists of the discussed preventive measures to avoid further progression.

### Single Osmotic Agents

Single osmotic agents to replace glucose that are currently available include the high molecular weight icodextrin and amino-acids that have a molecular weight somewhat less than that of glucose. The glucose poymer icodextrin should be used once daily for the long-dwell period in patients with kidney failure to prevent excessive accumulation of its metabolite maltose in the circulation. With this regimen plasma maltose concentrations average 2–3 mmol/L. In the absence of residual kidney function cellular uptake of this disaccharide occurs followed by degradation to glucose. Compared to glucose-based dialysis solutions the amount of intracellular glucose is very small, meaning that no cellular pseudohypoxia will be induced.

The use of amino-acids increases the nitrogen load, so they can only be administered in low concentrations for once-or twice daily administration. The osmotic effect of the commercially available 1.1% amino-acid solution is similar to that of a 1.5% glucose-based dialysis solution. Dialysis solutions with osmotic agents that are currently not commercially available include glycerol, xylitol and carnitine. These will be discussed in the following sections.

Glycerol has a molecular weight half that of glucose. It has been used in the past, but its high absorption can lead to hyperosmolality in the absence of residual kidney function (Matthys et al., 1987).

Xylitol is a polyol, i.e., a combination of a sugar and alcohol that is mainly used as artificial sweetener, for instance in chewing gum. Its application as osmotic agent has first been described 1982 in five patients (Bazzato et al., 1982). An increase of plasma uric acid concentration was the most important side effect and may explain why it was never applied on a large scale. Furthermore, the absorbed xylitol is oxidized to D-xylulose. NAD^+^ is consumed in this reaction, which means that in theory it can contribute to pseudohypoxia.

About 10 years ago another Italien group reported on L-carnitine as osmotic agent (Bonomini et al., 2011). This quarternary ammonium salt is mainly synthesized in the liver from lysine and methionine. The biological function of carnitine is mainly to facilitate the transport of fatty acids from the cytosol to the mitochondria where they are metabolized in the Krebs circle, which leads to NADH consumption and NAD^+^ generation. This may explain why the addition of L-carnitine to a dialysis solution improved the viability of fibroblasts *in-vitro* (Bonomini et al., 2011). The same study comprised a pilot in 4 PD patients who were administered a dialysis solution with L-carnitine as osmotic agent. It appeared that concentrations that were equimolar to glucose induced more ultrafiltration. This was likely caused by upregulation of AQP-1.

### Neutral pH, Low GDP Solutions

The so-called biocompatible dialysis solutions are all characterized by a low content of glucose degradation products and a neutral pH (L-GDP/N-pH solutions). They can be buffered with lactate, bicarbonate or combinations of both, but all contain glucose in similar concentrations as the conventional dialysis solutions. Therefore these solutions cannot be expected to have a pronounced effect on pseudohypoxia, although some modifying effect of the buffer substance may be possible. Morphologic studies comparing L-GDP/N-pH solutions with conventional ones showed indeed no effect on MMT (Del Peso et al., 2008), but some effects were present on pH related abnormalities and those induced by AGEs. The former include better mesothelial preservation, the latter consist of a reduced deposition of AGEs and less severe vasculopathy (Parikova et al., 2021). This finding supports the theory that GDPs enhance AGE formation (Jörres, 2003). Clinically the L-GDP/N-pH solutions are associated with reduced inflow pain and higher effluent CA 125 concentrations and with better preservation of small-pore fluid transport (Van Diepen et al., 2020). But, no effect was found on free water transport.

### Combinations of Osmotic Agents

In the absence of an ideal low molecular weight osmotic agent that can be employed for all exchanges combinations of osmotic solutes all in a low concentration are an attractive possibility. As the total osmotic effect of such solution is the sum of all individual ones, the required high value to induce adequate free water transport can be achieved. This hypothesis has been investigated in the GLAD study, which was done in rats with chronic kidney failure. (De Graaff et al., 2010). The animals received daily intraperitoneal administration of a hypertonic dialysis solution consisting of a mixture of 1.4% glycerol, 0.5% aminoacids and 1.1% dextrose (combined osmolality 512 mosmol/L) for 16 weeks. The GLAD solution showed better sodium sieving, less interstitial fibrosis and a lower vessel density, compared with animal exposed to a 3.86% dextrose (osmolarity 486 mosmol/L) solution. Replacing glucose-based dialysis solutions with various combinations of osmotic agents in PD patients with ultrafiltration failure for a short time already caused an improvement of ultrafiltration and sodium sieving, a lower VEGF, while an increase of effluent CA125 was found in a randomized clinical trial in stable PD patients (Zweers et al., 2001; Van Biesen et al., 2004; Smit et al., 2008).

A combination of carnitine and xylitol has been suggested to have a number of beneficial effects due to possible positive effects of the absorbed quantity. An *in-vitro* analysis using mesothelial and endothelial cells showed better viability of these cells compared to a glucose-based L-GDP/N-pH solution and a reduced expression of TGFβ and VEGF (Masola et al., 2021). Almost at the same time the results of a preliminary clinical study were published (Rago et al., 2021). In 10 patients with a PD duration of 6 months on average, the conventional PD solution was partly replaced with the carnitine/xylitol combination for 4 weeks. This was well tolerated and had no effect on peritoneal clearances and residual kidney function. A randomized multicenter clinical trial is currently in progress (Bonomini et al., 2021). It should be appreciated, however, that ref (Bonomini et al., 2021; Masola et al., 2021; Rago et al., 2021) were not published in any of the 72‐nephrology journals, but rather in journals owned by the same publisher of many online journals. This should not be regarded as disqualifying the scientific value of these cited papers, but it underlines the importance of the results of the multicenter clinical trial.

### Buffers in Dialysis Solutions

Lactate has traditionally been the buffer substance since more than 50 years. In theory the resulting high cytosolic lactate concentrations will inhibit the lactate dehydrogenase activity and thereby contribute to pseudohypoxia, but regrettably no clinical study has been published, comparing a lactate with a bicarbonate buffer, although one of the large dialysis companies sells L-GDP/N-pH solutions that are either buffered with lactate or with bicarbonate. The majority of studies in long-term PD patients have been performed with a solution containing bicarbonate and a low concentration of lactate. It is possible therefore that some of the beneficial effects of this bicarbonate/lactate buffered solution discussed above are due to some reduction of pseudohypoxia.

Replacement lactate by pyruvate is another possibility, because the absorbed pyruvate is directly metabolized in the Krebs circle generating NAD^+^. One *in-vivo* study has been published in rats that were exposed to daily administration of a 3.86% glucose-based dialysis solution either buffered with lactate or with pyruvate for a period of 20 weeks (van Westrhenen et al., 2008). Compared to the lactate group, the pyruvate exposed animals had a tendency to a lower plasma β-hydroxybutyrate/acetoacetate ratio, which suggests less general (pseudo) hypoxia. Histological examination showed reduced interstitial fibrosis and less severe vasculopathy without a difference in vascular density. Peritoneal small solute transfer was not different between the groups, but the pyruvate-exposed animals had better sodium sieving, suggesting better free water transport. It is a pity that pyruvate has never been investigated in PD patients, due to patent related issues.

### Additives to Dialysis Solutions

Many solutes can be added to PD solutions, mostly because they are absorbed from the peritoneal cavity to reach the circulation. Antibiotics are the best example. Protection of peritoneal tissues from damage caused by other components of the dialysis solution was the objective for the addition of the dipeptide alanyl-glutamine (Ala-Gln). This nutritional additive suppressed HIF-1 and collagen -1 levels in human peritoneal fibroblasts, cultured during hypoxia (Robertson et al., 2019). Amelioration of peritoneal fibrosis was also found in a murine PD model (Ferantelli et al., 2016). The addition of 8 mmol/L Ala-Gln to the dialysis solution in PD patients reduced effluent levels of methionine sulfoxide, a marker of oxidative stress (Wiesenhofer et al., 2019). The use of Ala-Glu addition to a L-GDP/N-pH dialysis solution in prevalent PD patients for 8 weeks was well tolerated and increased effluent CA 125 concentrations, suggesting better preservation of the mesothelium (Vychytil et al., 2018).

### Prophylaxis by Drugs

Possible targets include inhibition of TGFβ and of glucose transporters, and interference with the NADH/NAD^+^/ratio. Renal hypoxia upregulates the RAAS system and the protective effect of ACE inhibition has been well established in diabetic nephropathy (Lewis et al., 1993). Furthermore, an *in-vitro* analysis in cultured mesothelial cells showed involvement of the polyol pathway in the glucose mediated induction of TGFβ (Wong et al., 2003). Despite the extensive use of drugs that interfere with the RAAS system, only two clinical studies of their effects on peritoneal transport have been published (Kolesnyk et al., 2007; Kolesnyk et al., 2009). The first study was a single center analysis in 66 prevalent patients, of whom 36 used AII inhibitors. The control group showed an increase in small solute transfer after a follow-up of 3 years, which was absent in the patients on AII inhibition. No difference was present for parameters of fluid transport (Kolesnyk et al., 2007). Repeating the study in more than 200 patients of the NECOSAD (Netherlands cooperative study on the adequacy of dialysis) cohort confirmed the result on small solute transfer, analysis of fluid transport was impossible (Kolesnyk et al., 2009). These results are difficult to interpret and warrant a longer follow-up in more patients.

Despite its use in type-2 diabetes, no study has been published on possible peritoneo-protective effects of the PPAR-γ agonist rosiglitazone in PD patients. An analysis in a murine model of peritoneal exposure to dialysis solutions showed a reduction of fibrosis by rosiglitazone, a lower vascular density, and a reduced AGE deposition (Sandoval et al., 2010). Peritoneal transport parameters were not investigated.

The anti-estrogenic drug tamoxifen has fibrosis inhibiting properties as evidenced from its well-known effects in retroperitoneal fibrosis. Its use in EPS patients has been associated with better patient survival (Del Peso et al., 2003; Korte et al., 2011). A recent study in murine pancreatic tissue showed that tamoxifen regulated collagen cross-linking by inhibition of HIF-1 (Cortes et al., 2019), which is also supportive of the importance of pseudohypoxia in the genesis of peritoneal membrane alterations.

Inhibitors of glucose transporters comprise those of GLUT-1 and SGLT-2 inhibitors. No GLUT-1 inhibitor is currently available for use in humans (Reckeh and Waldmann, 2020). The presence of SGLT-2 has been shown in peritoneal mesothelial cells of humans and mice (Balzer et al., 2020). Their expression was upregulated by glucose containing dialysis fluids, which was prevented by SGLT-2 inhibition. However the significance of this observation is not evident, because active transport of sodium into the cell is the driving force for cellular glucose uptake (Navale and Paranjape, 2016) and mesothelial cells are unlikely to ingest sodium in large amounts. It is therefore no surprise that the administration of an SGLT-2 inhibitor had no effect on ultrafiltration in a rat PD model (Martus et al., 2021).

Interference with the NADH/NAD^+^ ratio is possible by interference with the polyol pathway by zopolrestat, an inhibitor of aldose reductase, which converts sorbitol to fructose. Administration of this drug in a long-term peritoneal exposure model in rats reduced peritoneal angiogenesis and fibrosis (van Westrhenen et al., 2005). However this drug is not available for use in humans, because of side-effects. The oral administration of nicotinamide mononucleotide, a precursor of NAD^+^, is becoming increasingly popular in Japan to prevent age–related disorders, like diabetes and cardiovascular disease. It is based on the observation that aging is accompanied by decreased cellular NAD^+^ levels. Most studies have been experimental ones, as discussed recently in a paper that also summarized ongoing studies in humans suffering from various conditions (Hong et al., 2020). Administration of a single dose in healthy men showed no side effects (Irie et al., 2020) and it is sometimes available as a nutritional supplement. No experimental or clinical study in PD has been published, but further exploration of nicotinamide mononucleotide administration in PD is attractive.

## Summary and Conclusion

The functional and morphological alterations that occur in the peritoneal dialysis membrane after long-term PD can be ascribed to glucose-induced formation of AGEs causing vasculopathy, and to pseudohypoxia, defined as an increased cellular NADH/NAD^+^ ratio. The latter causes upregulation of HIF-1, which leads to upregulation of GLUT-1 and its expression on peritoneal cells, and to upregulation of the growth factors TGFβ, VEGF, PAI-1 and CTGF. GLUT-1 in the cell membrane increases the influx of glucose and induces a vicious circle leading to a progressive decline of free water transport. The growth factors stimulate fibrosis and angiogenesis. The obvious therapeutic approach consists of combinations of various osmotic agents, all in low concentrations. This reduces the toxicity of all individual compounds while their osmolalaties add up, maintaining their over-all osmotic effects. All other discussed options are secondary.
