# Corrigendum: Aging of the Peritoneal Dialysis Membrane

**DOI:** 10.3389/fphys.2022.950933

**Published:** 2022-08-24

**Authors:** Raymond T. Krediet

**Affiliations:** Division of Nephrology, Department of Medicine, Amsterdam University Medical Center, Amsterdam, Netherlands

**Keywords:** peritoneal dialysis, ultrafiltration failure, glucose, pseudohypoxia, GLUT-1, growth factors, osmotic agents

In the original article, there was an error. A remark at the end of **Combinations of Osmotic Agents** could be interpreted as an expression of criticism of the scientific content of specific cited papers, which was never the intention.

A correction has been made to **Prevention and Treatment**, **Combinations of Osmotic Agents**, final sentence. The corrected sentence appears below:

It should be appreciated, however, that ref (Bonomini et al., 2021; Masola et al., 2021; Rago et al., 2021) were not published in any of the 72-nephrology journals, but rather in journals owned by the same publisher of many online journals. This should not be regarded as disqualifying the scientific value of these cited papers, but it underlines the importance of the results of the multicenter clinical trial.

The author apologizes for this error and states that this does not change the scientific conclusions of the article in any way. The original article has been updated.

